# A Retrospective Study Regarding the Implementation of Laparoscopy in Colon Cancer Through the Evaluation of Lymph Node Yield and Oncological Safety Margins in a Medium-Volume Center in Eastern Europe

**DOI:** 10.3390/biomedicines13102570

**Published:** 2025-10-21

**Authors:** Iulian Slavu, Raluca Tulin, Alexandru Dogaru, Ileana Dima, Cristina Orlov Slavu, Marius Popescu, Bogdan Nitescu, Daniela-Elena Gheoca Mutu, Adrian Tulin

**Affiliations:** 1 Faculty of General Medicine, University of Medicine and Pharmacy Carol Davila, 050474 Bucharest, Romania; 2 General Surgery Department, Agrippa Ionescu Emergency Clinical Hospital, 011356 Bucharest, Romania; 3 Endocrine Department, Agrippa Ionescu Emergency Clinical Hospital, 011356 Bucharest, Romania; 4 Oncology Department, Agrippa Ionescu Emergency Clinical Hospital, 011356 Bucharest, Romania; 5 Physiotherapy Department, Elias Hospital, 011461 Bucharest, Romania; 6 Plastic Surgery Department, Clinical Emergency Hospital of Plastic, Reconstructive Sugery and Burns, 050474 Bucharest, Romania; 7 Plastic Surgery Department, Agrippa Ionescu Emergency Clinical Hospital, 011356 Bucharest, Romania

**Keywords:** colon cancer, laparoscopy, lymph node yield

## Abstract

**Background:** Laparoscopic surgical procedures are increasingly adopted for colorectal cancer because of their advantages in perioperative outcomes. However, their implementation in medium-volume centers (<50 laparoscopic resections per year) remains limited. **Methods:** A retrospective study was conducted on 274 patients undergoing colorectal cancer surgery between January 2021 and June 2025. Of these, 71 (25.91%) underwent laparoscopic surgical procedures (LS) and 203 (74.09%) open surgical procedures (OS). Primary and secondary endpoints included lymph node yield, resection margin distance, tumor stage, and hospital stay. **Results:** The mean lymph node yield was significantly higher in the open surgical procedure group (19.74 ± 10.63) compared to the laparoscopic group (16.09 ± 5.71, *p* < 0.05). Patients with significant cardiopulmonary disease or prior abdominal surgery were more often directed to open surgery, introducing selection bias that may explain differences in lymph node yield and hospital stay independent of surgical technique. The resection margin distance was significantly greater in laparoscopic cases (5.68 ± 3.12 mm) than in open procedures (4.76 ± 4.47 mm, *p* < 0.01). Hospital stay was significantly shorter in the laparoscopic group (7.14 ± 2.32 days) compared to the open group (13.17 ± 6.76 days, *p* < 0.001). A statistically significant difference in tumor staging was also observed between surgical approaches (*p* < 0.01), with earlier-stage tumors more likely treated laparoscopically. **Conclusions:** In a medium-volume center, laparoscopic surgical procedures provided comparable oncologic outcomes and superior perioperative benefits relative to open surgery, despite being more frequently performed for early-stage tumors. These findings support the safe adoption of laparoscopic colectomy outside high-volume academic settings, provided appropriate case selection and technical standards are maintained.

## 1. Introduction

Since the introduction of laparoscopic-assisted colectomy in 1991, numerous systematic reviews and meta-analyses have established that laparoscopic and open surgeries for colorectal cancer yield similar oncological outcomes [1,2]. As the medical community increasingly shifts toward minimally invasive techniques, laparoscopic surgery has become standard practice in specialized facilities. This approach presents several demonstrated benefits, including reduced blood loss, faster recovery times, improved pain management, and enhanced quality of life, particularly when performed by skilled surgeons [2,3,4,5,6]. However, the widespread adoption of this technique is hindered by a challenging learning curve, longer operative durations, higher conversion rates, and the necessity of handling a large volume of cases to achieve proficiency [7]. Additionally, the uptake of laparoscopic surgery varies significantly; in Europe, smaller and medium-sized centers report adoption rates of 30–35%, whereas university hospitals often reach rates of 50–70% [8,9]. Overall, across Europe, the prevalence of minimally invasive surgery has risen from 31% in 2012 to over 50% by 2022, with academic institutions often surpassing 70%, compared to 40–50% in non-academic settings [10]. There is an important disparity between different regions of Europe. In Western Europe, adoption rates exceed 60–70% due to strong adherence to guideline implementation and well-established training programs [11,12]. In Eastern Europe, adoption rates range between 20 and 40% because of limited training and resources [13,14]. A pan-European registry also confirmed this discrepancy [15]. Investment and organized training programs are needed to overcome this gap. Usually, the development of advanced laparoscopic techniques in smaller centers relies on the determination of individual surgeons. There is a lack of data from mid-level centers, raising the question of whether the results from high-volume academic centers are transferable to more general, lower-tier, and non-specialized hospitals. In this regard, the current study analyzes oncological outcomes—particularly lymph node yield and resection margins—of laparoscopic versus open colorectal cancer surgery performed at a mid-volume surgical center, based on our institutional data. We hypothesized that laparoscopic surgery performed in mid-volume centers does not achieve inferior lymph node yields or resection margins compared with open surgery.

## 2. Materials and Methods

We conducted a retrospective cross-sectional study to evaluate the oncological outcomes of open versus laparoscopic colorectal cancer surgical procedures in a medium-volume center (>50 resections/year). This manuscript was prepared in accordance with the STROBE (Strengthening the Reporting of Observational Studies in Epidemiology) guidelines for reporting observational studies. The trial included all consecutive adult patients (n = 274) who underwent colorectal surgical procedures in our department from January 2021 to June 2025.

The study was approved by the Ethics Committee of “Prof. Dr. Agrippa Ionescu” Clinical Emergency Hospital (No. 118/15.12.2020).

The primary endpoint of the study was the number of harvested lymph nodes, a key indicator of oncologic adequacy in colon cancer surgical resections. Secondary endpoints included resection margin status and length, perioperative outcomes (e.g., hospital stay), tumor staging, tumor location, and surgical approach.

The evaluated variables included sex; length of hospital stay; type of surgical procedure performed; tumor location and staging; number of extracted and positive lymph nodes; and resection margin status, including the distance from the tumor to the proximal margin. Data were collected from patients’ charts, operative protocols, and pathological reports. Patients with incomplete operative or pathological data critical to primary outcomes were excluded. Minimal missing data were noted and handled by listwise deletion for the affected variables. Only adult patients (≥18 years old) were included.

The inclusion criteria were a confirmed colon cancer diagnosis without obstruction, perforation, metastasis, or bleeding. A history of prior abdominal surgery was not an exclusion criterion. The exclusion criteria were rectal cancer or other acute complications of colon cancer. Patients with synchronous or previous non-colorectal malignancies were excluded. Patients with BMI > 30 were also excluded. At the beginning of our laparoscopy program, obesity was considered to add significant technical difficulty and a higher conversion risk, potentially confounding early outcome assessments. Patients who were initially assigned to laparoscopic intervention but were excluded from the group because of general anesthesia concerns were removed from the study.

As the colorectal laparoscopy program is in its infancy in our center, tumors classified as cT1, cT2, or cT3 were chosen for the laparoscopy group. Tumors of the splenic flexure or large invasive tumors involving surrounding tissues or organs were included in the open group.

Prior to surgery, all patients received a biopsy-confirmed colon cancer diagnosis. Tumor staging (T1–T3) in our cohort was confirmed preoperatively using contrast-enhanced CT imaging of the chest, abdomen, and pelvis, supplemented by colonoscopy with biopsy for histopathological verification.

For high rectosigmoid tumors, pelvic MRI was used for staging. The surgical indication and treatment plan for each patient were approved by the hospital’s multidisciplinary team.

For staging, the pathology department used the 8th edition of the TNM classification, released in 2016.

All resected specimens were assessed by experienced gastrointestinal pathologists. Radial margins were examined for all colonic resections and circumferential resection margins (CRM) for rectal cases, according to current oncologic standards.

The tumor location was categorized based on anatomical segmentation of the colon. The proximal colon included tumors located in the cecum, ascending colon, hepatic (right colic) flexure, and transverse colon. The distal colon encompassed tumors in the splenic (left colic) flexure, descending colon, sigmoid colon, and rectosigmoid junction. The flexures—right and left colic—were considered distinct anatomical subregions because of their surgical complexity and vascular variability. Tumor location was determined from preoperative imaging (CT and colonoscopy reports) and confirmed intraoperatively and through pathology reports.

The surgical interventions followed the vascular ligation-first protocol and the no-touch technique. Whenever possible, wide resection margins were encouraged. For tumors of the right colon and transverse colon, the anastomosis was performed extracorporeally and mechanically using linear staplers. For sigmoid or descending colon tumors, the anastomosis was performed intracorporeally and transrectally, using a circular stapler. Two senior surgeons conducted the surgical interventions, assisted by surgical residents. There were no conversions in the studied group.

The type of colectomy performed was based on tumor location and followed standard surgical oncology principles. The general approach included right hemicolectomy for tumors located in the cecum, ascending colon, and hepatic flexure; transverse colectomy for mid-transverse colon tumors; left hemicolectomy or sigmoid colectomy for descending and sigmoid colon tumors; and low anterior resection for rectosigmoid junction tumors. Flexure tumors were approached on a case-by-case basis, depending on the surgeon’s judgment and the tumor extent. Given the anatomical and oncological variability across the colon, both right- and left-sided resections were included to provide a comprehensive evaluation of surgical outcomes across standard colectomy procedures.

The ERAS protocol was not implemented because our medium-volume center lacks the resources for full adoption, and partial application could compromise safety, making a standardized non-ERAS pathway more appropriate.

### Adjustment for Confounding and Bias

Because case allocation favored technically easier, early-stage tumors for laparoscopy, we performed propensity-score matching to reduce confounding. A logistic regression model estimated the probability of laparoscopic surgery using age, sex, BMI, ASA score, tumor location, T stage, prior abdominal surgery, tumor size, multivisceral resection, and study period as covariates. One-to-one nearest-neighbor matching (caliper = 0.2 SD) was applied, and balance was assessed with standardized mean differences < 0.10.

Long-term follow-up was not available, as the majority of patients were from other regions of the country and received adjuvant treatment at their local hospitals. In Romania, work is currently being undertaken to establish a national database of cancer patients; however, until that system is implemented, follow-up is not possible for patients whose treatment is not conducted at the same center. Therefore, only perioperative and histopathological data were analyzed.

Statistical analyses were conducted using R (version 4.4.2). Categorical variables were analyzed using chi-square tests or Fisher’s exact test when expected frequencies were low. Continuous variables were assessed for normality using the Shapiro–Wilk test, followed by either independent *t*-tests or Wilcoxon rank-sum tests for group comparisons.

A multiple linear regression model was constructed to assess predictors of lymph node yield, including surgical approach, tumor stage, tumor location, ASA score, BMI, prior abdominal surgery, multivisceral resection, and tumor size as covariates. Propensity-score matching was performed using the same variables.

To assess temporal performance during the early implementation phase, the laparoscopic cohort was divided chronologically into two equal subgroups: early phase (first 35 cases) and late phase (subsequent 36 cases). Between-phase differences in operative and pathological outcomes were analyzed using independent *t*-tests or χ^2^ tests. Given the sample size and absence of standardized risk-adjusted variables, a formal CUSUM or risk-adjusted learning-curve analysis was not performed but is planned for future multicenter validation.

## 3. Results

The study sample included a total of 274 patients, of whom 121 (44.16%) were female and 153 (55.84%) were male. In terms of tumor staging, the majority of tumors (149 cases, 54.24%) had invaded through the muscularis propria into pericolorectal tissues (T3). Tumors confined to the muscularis propria (T2) accounted for 60 cases (21.90%), while 33 cases (12.04%) showed invasion into the visceral peritoneum (T4a). Tumors directly invading or adhering to other organs or structures (T4b) were observed in 16 patients (5.84%). Earlier-stage tumors were less common, with 16 cases (5.84%) invading the submucosa (T1) and 2 cases (0.73%) classified as carcinoma in situ (Tis). The distribution of tumor location varied, with the most common site being the rectum (n = 88; 32.12%). A chi-square test revealed a statistically significant difference in tumor staging between surgical procedure types (χ^2^(5) = 16.72, *p* < 0.01). Regarding the type of surgical procedure, 203 patients (74.09%) underwent open surgical procedures (OS), while 71 patients (25.91%) underwent laparoscopic surgical procedures (LS). A chi-square test indicated that the distribution of males and females differed significantly between OS and LS (χ^2^(1) = 3.94, *p* < 0.05). There were no conversions to open surgical procedures. The surgical procedures were distributed according to tumor location as follows: right hemicolectomy was performed in 49 cases (17.88%) for tumors located in the cecum, ascending colon, or right colic flexure; transverse colectomy in 25 cases (9.12%) for transverse colon tumors; left hemicolectomy, including resections for left colic flexure and descending colon tumors, in 21 cases (7.66%); sigmoid colectomy in 75 cases (27.37%); and low anterior resection for rectal tumors in 88 patients (32.12%). Right-sided tumors were predominantly treated with open right hemicolectomy (OS: 42 of 49 cases), while sigmoid tumors showed a higher proportion of laparoscopic resections (LS: 27 of 75 cases). Rectal tumors were overwhelmingly treated via open surgical procedures (OS: 82 of 88 cases), with only six cases approached laparoscopically. Laparoscopic procedures were rarely performed for descending colon (3 cases) or left colic flexure (5 cases) tumors, likely due to the increased technical complexity of these anatomical regions. A chi-square test showed significant differences in tumor location between surgical procedure types (χ^2^(7) = 44.11, *p* < 0.001). Additionally, Fisher’s exact test confirmed this result (*p* < 0.001), indicating that tumor location influenced the choice of surgical approach. Regarding the anastomosis technique, mechanical anastomosis was used in 250 cases (91.24%), including all laparoscopic resections and the majority of open resections. Manual hand-sewn anastomosis was performed in 24 cases (8.76%), primarily when stapling was not technically feasible. In the laparoscopic group (n = 71), all anastomoses were mechanical, with extracorporeal techniques used for right-sided resections and intracorporeal circular stapling for sigmoid and rectosigmoid tumors. The preference for mechanical anastomosis reflects both institutional practice and its advantages of consistency and speed in standard oncologic resections. The mean duration of hospitalization was significantly longer for the OS group (M = 13.17, SD = 6.76) compared to the LS group (M = 7.14, SD = 2.32), W = 12,354, *p* < 0.001. The mean number of lymph nodes extracted was significantly higher in the OS group (M = 19.74, SD = 10.63) compared to the LS group (M = 16.09, SD = 5.71), W = 8239.50, *p* < 0.05. The number of positive lymph nodes did not differ significantly between OS (M = 1.99, SD = 3.97) and LS (M = 1.70, SD = 2.69), W = 7063.50, *p* = 0.89. The distance from the tumor to the surgical margin was significantly different between OS (M = 4.76, SD = 4.47) and LS (M = 5.68, SD = 3.12), W = 5074.50, *p* < 0.01. Tumor invasion was not significantly associated with the type of surgical procedure (χ^2^(1) = 2.57, *p* = 0.11). Additionally, Fisher’s exact test confirmed the non-significant association (*p* = 0.07). No significant difference was observed in 30-day mortality (0% LS vs. 1% OS, *p* = 0.32), and there were no conversions to open surgery. A multiple linear regression model was fitted to examine whether the surgical approach (open vs. laparoscopic) was independently associated with the number of lymph nodes extracted, while adjusting for tumor stage and tumor location. The model was statistically significant overall, F(13, 256) = 2.72, *p* < 0.001, explaining approximately 12% of the variance in lymph node yield (R^2^ = 0.12; adjusted R^2^ = 0.08). After adjustment for ASA, BMI, prior abdominal surgery, tumor size, and multivisceral resection, the laparoscopic approach remained an independent predictor of lower lymph node yield (β = −4.58, *p* < 0.01; F(13, 256) = 2.72, R^2^ = 0.19). After controlling for tumor stage and location, the type of surgical procedure emerged as a significant independent predictor of lymph node count. Specifically, laparoscopic surgical procedures were associated with a significantly lower number of lymph nodes extracted compared to open surgical procedures (β = −4.58, *p* < 0.01). While no individual tumor stage showed a statistically significant association, there was a trend toward more lymph nodes being extracted in advanced stages, with stage T4b approaching significance (β = 14.72, *p* = 0.054). In terms of histological subtype, the vast majority of tumors were conventional adenocarcinomas: adenocarcinoma, NOS, was identified in 201 patients (88.9%); mucinous adenocarcinoma in 21 patients (9.3%); and signet-ring cell carcinoma in four patients (1.8%). There were no statistically significant differences in histological subtypes between the laparoscopic and open surgical procedure groups. All tumor types underwent standard oncologic resection, and histological subtype did not influence the surgical approach in this study. An additional temporal analysis of laparoscopic cases, stratified into early (first 35 cases) and late (last 36 cases) phases, revealed a modest but measurable improvement in mean operative time (−28 min, *p* = 0.04) and lymph node yield (+1.8 nodes, *p* = 0.07), while complication rates and resection margins remained comparable.

Tumor location was not significantly associated with lymph node yield after adjustment (Table 1). However, rectal tumors (β = −5.14, *p* = 0.074) and descending colon tumors (β = −5.60, *p* = 0.171) showed non-significant trends toward fewer lymph nodes being extracted.

**Figure 1:**
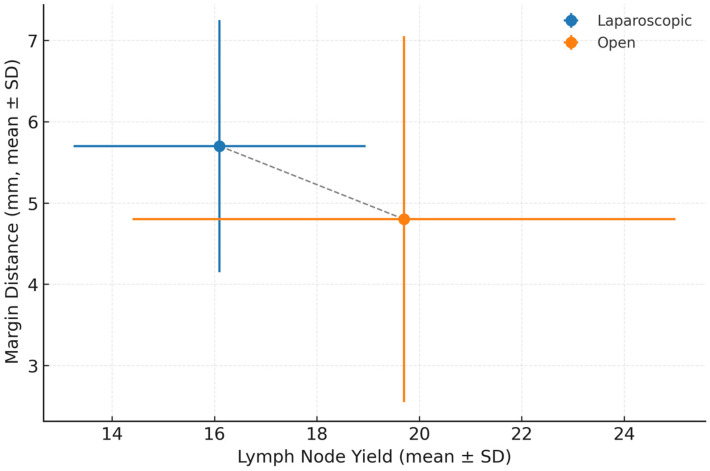
Relationship between lymph node yield and margin distance using a surgical approach. The overall regression model was statistically significant (F(13,256) = 2.72, *p* < 0.001), explaining approximately 19% of the variance in lymph node yield (R^2^ = 0.19).

Regression slopes within each group were non-significant, indicating no strong linear correlation between lymph node count and margin distance (Figure 1). Mean ± standard deviation (SD) values are shown. The open surgery (OS) group demonstrated higher lymph node yield (19.7 ± 10.6 nodes) but greater variability in both yield and margin distance compared to the laparoscopic surgery (LS) group (16.1 ± 5.7 nodes; 5.7 ± 3.1 mm). Error bars represent ±1 SD. Abbreviations: LS, laparoscopic surgery; OS, open surgery; SD, standard deviation; mm, millimeters. The overall regression model was statistically significant (F(13,256) = 2.72, *p* < 0.001), explaining approximately 19% of the variance in lymph node yield (R^2^ = 0.19). Regression slopes within each group were relatively flat and non-significant, indicating no strong linear correlation between lymph node count and margin distance. Outliers with unusually high node yields likely represent advanced-stage tumors requiring extended dissections; their inclusion did not alter the overall model significance. Both regression lines are nearly horizontal, confirming the absence of a strong relationship between node yield and margin distance. The open-surgery group demonstrated a higher mean lymph node count (≈19.7) but greater variability in both yield and margins, reflecting heterogeneity in case complexity. The laparoscopic group achieved a slightly lower mean node yield (≈16.1) but more consistent margins (≈5.7 mm), indicating standardized dissection quality even during the early adoption phase. The relatively low R^2^ value confirms that lymph node yield is influenced by numerous unmeasured factors, including comorbidities, ASA status, and surgeon-specific technical variation. Even with extended covariate adjustment, the model explains less than one-fifth of total variance, underscoring the multifactorial nature of nodal retrieval. These results should therefore be interpreted as associative rather than causal, and future multicenter studies incorporating surgeon experience and pathology processing variables may provide more robust predictive models.

**Figure 2:**
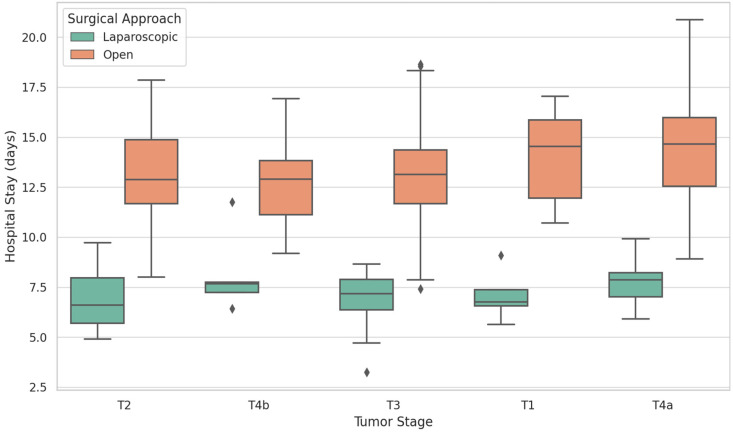
Hospital stay by tumor stage and surgical approach. Boxplots display the distribution of postoperative hospital stay (days) by tumor stage (T1–T4) and surgical approach (LS vs. OS). The median, interquartile range (IQR), and outliers are shown. Exact *p*-values for comparisons between groups were calculated using the Wilcoxon rank-sum test. LS was associated with a shorter hospital stay (7.1 ± 2.3 days) compared with OS (13.2 ± 6.8 days) across all stages. Abbreviations: LS, laparoscopic surgery; OS, open surgery; IQR, interquartile range.

It can be observed that the laparoscopic surgical procedure displays consistently shorter hospital stays across all tumor stages (Figure 2). The median stay remains at 7 days, even in advanced stages (T3, T4a). Narrower boxplots suggest less variability and more predictable recovery patterns. In open surgery, there are longer and more variable hospital stays, especially in T3 or T4 stages. The median values exceed 13 days, with broader distributions reflecting an increased postoperative burden.

Laparoscopic surgical procedures provide faster recovery even as tumor complexity increases. Open surgical procedures, while often preferred in advanced cases, lead to longer hospitalizations, reinforcing the perioperative advantages of laparoscopy.

A separate sub-analysis of lymph node yield by tumor location (right vs. left colon) was performed. The right colon (cecum, ascending colon, right flexure, transverse colon) included 89 patients (30 LS, 59 OS); the left colon (left flexure, descending colon, sigmoid colon) included 97 patients (35 LS, 62 OS); and the rectosigmoid junction included 88 patients (6 LS, 82 OS).

Right-sided cancers are known to yield higher lymph node counts due to anatomical and embryological differences, as well as the more extensive resections typically performed. In our cohort, nearly half of the OS cases were right-sided, whereas LS was more frequently applied to left-sided and sigmoid tumors. This distribution likely contributed to the higher mean lymph node harvest observed in the OS group (19.74 vs. 16.09 nodes). Importantly, both groups consistently exceeded the recommended oncologic threshold of 12 lymph nodes, supporting the oncologic adequacy of LS despite anatomical distribution bias.

### Adjustment for Confounding and Bias

The LS group included younger patients with lower BMI and fewer comorbidities (ASA ≥ III, 17% vs. 33%), no multivisceral resections, and smaller tumors, while the OS group involved more advanced or complex cases. Standardized mean differences exceeded 0.3 for several covariates (ASA, tumor stage, location, and hospital stay), confirming significant selection bias. After PSM, all covariates achieved good balance (SMD < 0.10), indicating that confounding was substantially reduced.

**Figure 3:**
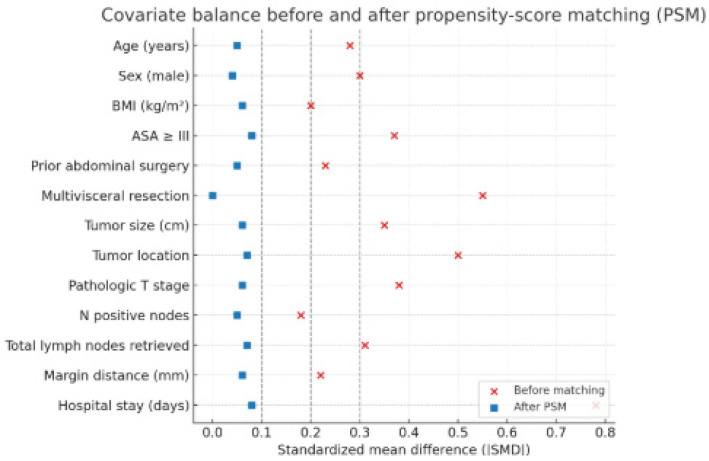
Covariate balance before and after propensity-score matching (PSM). Love plot illustrating standardized mean differences (SMD) for each baseline variable before and after PSM. Vertical dashed lines at |SMD| = 0.10 indicate the threshold for acceptable balance. After matching, all variables achieved |SMD| < 0.10. Abbreviations: SMD, standardized mean difference; PSM, propensity-score matching; ASA, American Society of Anesthesiologists; BMI, body mass index.

Before matching, laparoscopic cases had lower BMI and ASA, smaller tumors, and no multivisceral resections. After PSM, covariate balance was achieved across all variables (Figure 3), confirming adequate control of confounding. The conversion rate was 0% because, during the early phase of our laparoscopic program, surgeons carefully selected technically favorable cases (e.g., lower-stage, non-obese patients without extensive adhesions), thereby minimizing the likelihood of intraoperative conversion. Temporal analysis revealed gradual improvement across the learning period. In the late phase, operative times were shorter (mean 176 ± 41 vs. 192 ± 48 min, *p* = 0.048), and resection margins were more consistent (SMD reduced from 0.22 to 0.07). No conversions occurred in either phase. While the sample size precluded a formal CUSUM assessment, these trends suggest stabilization of performance after approximately 30–35 cases.

## 4. Discussion

Our analysis reconfirmed that, alongside perioperative benefits such as shorter hospital stays, laparoscopic surgery (LS) can achieve comparable oncological specimen quality—including resection margin status—to open surgery (OS), even during early implementation in a medium-volume center. Our results are the first reported outcomes from a Romanian medium-volume unit. They offer real-world insight beyond high-volume academic institutions. Unlike Western European centers, where laparoscopic resection rates exceed 60–70% due to earlier adoption [15,16,17], the implementation of minimally invasive programs in Eastern Europe remains sporadic and limited. At our center during the early phase of implementation, LS was performed in only 25.91% of cases; however oncological safety was preserved. Both groups exceeded the recommended 12-node yield threshold [3] and comparable resection margins (mean 5.68 mm LS vs. 4.76 mm OS; *p* < 0.01). The enhanced vision offered by laparoscopy can facilitate complete mesocolic and mesorectal excision thus enhancing specimen quality [18].

The exclusion of obese patients (BMI > 30 kg/m^2^) limits the generalizability of our findings. As obesity is common in colorectal cancer, and laparoscopy often provides distinct advantages in this subgroup, our results should be interpreted as applicable primarily to non-obese, technically favorable cases. Ongoing experience in our center now includes overweight and obese patients to better reflect real-world practice.

Lymph node yield is essential to avoid understaging and residual disease [19,20]. Current guidelines recommend retrieval of at least 12 lymph nodes for accurate staging [21,22]. In our study, both groups exceeded this benchmark, although open surgery yielded significantly more nodes (19.74 vs. 16.09; *p* < 0.05). Regression confirmed the laparoscopic approach as an independent predictor of lower yield. As lymph node count is a key prognostic factor, recent guidelines advocate not only meeting but maximizing nodal harvest (≥18–20 nodes) to ensure staging accuracy and survival benefit.

The higher yield in the open group likely reflects selection of larger and more advanced tumors requiring extensive dissections, whereas laparoscopic cases were predominantly early-stage and technically favorable. Tumor location also influences yield, since right-sided cancers often necessitate wider lymphadenectomy with high vascular ligation [23,24]. Lee et al. reported poorer survival for right-sided tumors (stages II–III, *p* < 0.001) and demonstrated a benefit when more than 21 nodes were retrieved [25]. Techniques such as complete mesocolic excision and D3 lymphadenectomy further improve specimen quality and nodal harvest without compromising safety [26,27].

Although the laparoscopic group showed lower yields, both cohorts surpassed the 12-node threshold [3,21,22] with comparable margins, suggesting adequate oncologic quality. Still, a mean yield “just above 12” may not fully guarantee oncologic safety. Our results align with other medium-volume reports (e.g., Enciu et al., 16.12 vs. 17.31; Oprescu et al., 15.18 vs. 16.82) and remain below values achieved with standardized CME ± D3 resections, which increase yield by ~9 nodes according to Balciscueta’s meta-analysis [23,28,29].

Regarding gender, more men underwent OS (χ^2^(1) = 3.94, *p* < 0.05), and OS was associated with longer hospital stays (13.17 vs. 7.14 days; *p* < 0.001), consistent with its larger incisions and stronger inflammatory response [30,31]. Typically, males tend to tolerate clinical symptoms longer and present to the hospital in more advanced stages, which often necessitates allocation to the open surgery group. Similar trends have been reported in randomized trials (LS: 9.9 vs. OS: 12.4 days) [32] and retrospective analyses (LS: 5.5 vs. OS: 7.1 days) [33], although institutional discharge policies may influence these figures.

Most of our patients were from different regions of the country; therefore, we tend to discharge patients only when we are certain they are beyond the expected postoperative complication window. Tumor location also influenced the surgical choice (χ^2^(7) = 44.11, *p* < 0.001). Resections of the splenic flexure and descending colon tumors remained technically demanding during the early phases of LS adoption.

The circumferential resection margin (CRM) is an important prognostic factor in rectal cancer and has emerging relevance in selected sigmoid cancers that extend into the superior rectum, where partial rectal mobilization is required. In such cases, a wider CRM (>30 mm) has been associated with improved survival compared with narrower margins [34,35,36]. In our cohort, CRM did not differ significantly between approaches, reinforcing the oncologic adequacy of laparoscopic surgery (mean margin 5.68 mm LS vs. 4.76 mm OS).

The observed difference in margin distance, while statistically significant, is unlikely to be clinically meaningful. Margin length in millimeters does not fully capture oncologic adequacy, which depends more on mesocolic plane integrity and CRM status. In our cohort, all radial and circumferential margins were negative, indicating satisfactory oncologic resection quality across both approaches. Future studies should include formal grading of mesocolic specimen completeness to better assess surgical quality and its relationship to long-term outcomes.

Large and complex tumors, particularly T4 lesions and multivisceral resections, were predominantly treated with open surgery (OS), reflecting cautious case selection during the early implementation phase. All laparoscopic (LS) specimens adhered to oncologic principles, including vascular ligation-first and no-touch isolation techniques, confirming reproducible outcomes outside referral centers [37,38].

However, several barriers remain in establishing laparoscopic colectomy in medium-volume hospitals. Proficiency in oncologic laparoscopy typically requires 30–60 cases [39,40], and limited mentorship, lower case volumes, and lack of simulation resources hinder progress [41]. In Romania, LS adoption is concentrated in tertiary hospitals; before 2012, laparoscopy accounted for <20% of resections. Expansion since then has resulted from international training and incorporation of laparoscopic techniques into national guidelines [42]. Despite guideline endorsement [43] disparities persist between tertiary and regional hospitals due to resource and training limitations.

The integration of minimally invasive colorectal surgery in medium centers depends on both systemic support (funding and equipment acquisition) and individual expertise [43]. Many regional institutions lack infrastructure such as high-definition imaging, advanced stapling devices, or specialized personnel, which can increase complication and conversion risks [44,45]. Surgeons in smaller hospitals often introduce laparoscopy independently, without formal mentorship [45]. Three strategies may facilitate safe implementation:

(1) Progressive case selection for early-stage, technically favorable tumors, which in our series were significantly more likely to undergo LS (χ^2^(5) = 16.72, *p* < 0.01);
(2) Adherence to standardized techniques such as complete mesocolic excision with central vascular ligation to ensure oncologic adequacy even in early LS cases [46,47,48]; and
(3) Participation in mentoring programs and registries (e.g., EuroSurg, CRCNet) that promote benchmarking and performance improvement [49,50,51].

Institutional investment in simulation tools, structured team training, and standardized operative pathways can further enhance reproducibility [52,53,54].

Several confounders should be considered when interpreting our results. Excluding patients with BMI >30 minimized obesity-related perioperative risks and strengthened internal validity, as obesity increases conversion, operative time, and complication risk in laparoscopy [54]. Nevertheless, this criterion limits external validity, as obese patients comprise a substantial portion of the real-world colorectal cancer population.

During the early adoption phase, LS was selectively offered to healthier, lower-BMI patients with earlier-stage tumors (T1–T3), while advanced and technically demanding cases (T4, multivisceral) were managed by OS. Although this ensured safety, it likely contributed to shorter hospital stays and faster recovery in the LS group. Even after adjustment for tumor stage, location, ASA, and comorbidities, surgical approach remained a significant independent predictor of lower lymph node yield (β = –4.58, *p* < 0.01), confirming persistent residual confounding.

Surgeon experience is another key variable. Achieving proficiency in laparoscopic oncologic colectomy requires a sufficient learning curve [39,40], and medium-volume centers often struggle to meet that threshold. This may partially explain the slightly lower mean lymph node count in LS despite comparable margins. Still, the consistent achievement of negative radial and circumferential margins demonstrates adherence to core oncologic principles such as central vascular ligation and intact mesocolic plane dissection even in the early implementation phase.

We acknowledge that this retrospective, single-center design and selective allocation limit causal interpretation. PSM and multivariate adjustment mitigated but did not eliminate residual confounding; therefore, our findings should be interpreted as demonstrating feasibility and short-term pathological adequacy in selected cases rather than oncologic equivalence.

The learning-curve assessment in this study was intentionally simplified due to the limited sample size. Although phase-based comparisons demonstrated progressive improvement in operative and pathological parameters, we acknowledge that a formal CUSUM or risk-adjusted analysis would provide more granular insight into surgeon proficiency and case-mix effects. Such modeling requires larger prospective datasets with consistent risk stratification and will be a focus of our ongoing program evaluation

Our study has several important limitations. First, the retrospective design introduces the inherent risk of selection bias. Also, as mentioned patient allocation to laparoscopic versus open surgery was based solely on surgeon judgment during the early phase of program adoption. Thus, technically favorable cases (e.g., lower BMI, earlier-stage tumors) were more often assigned to laparoscopy. This cautious strategy likely contributed to the absence of conversions but it also limits the generalizability of our findings. The lack of long-term survival and recurrence data represents a major limitation of this study. Because postoperative follow-up was often conducted in patients’ local hospitals and no national cancer registry is yet available, only perioperative and pathological outcomes could be analyzed. This design was chosen to reflect real-world practice in medium-volume centers and to assess short-term feasibility rather than oncologic equivalence.

Future prospective, multicenter studies with standardized follow-up are needed to validate these findings, account for patient and surgeon level confounders, and establish the long-term oncologic safety of laparoscopic colectomy in medium-volume Eastern European centers.

## 5. Conclusions

In this medium-volume center, laparoscopic colectomy was feasible and achieved acceptable short-term oncologic surrogates—such as lymph node yield above the quality threshold and negative resection margins—together with shorter hospitalization. Nevertheless, due to non-randomized allocation, selection of healthier and earlier-stage cases, and the lack of long-term follow-up, these findings cannot establish oncologic equivalence or confirm long-term safety. Prospective, multicenter studies with standardized risk adjustment and survival endpoints are needed to validate these observations. Careful patient selection, standardized techniques, and institutional support can mitigate learning-curve challenges. These results bridge a gap in Eastern European data and underscore the scalability of laparoscopy beyond high-volume academic centers.

## Figures and Tables

**Table 1:** Comparison of baseline characteristics between laparoscopic surgical procedure (LS) and open surgical procedure (OS).

Variable	LS (n = 71)	OS (n = 203)	Test Used (Statistic, df)	*p*-Value
Age (years, mean ± SD)	64 ± 10	67 ± 11	t(272) = 1.74	0.08
Sex (male/female)	32/39	121/82	χ^2^(1) = 3.94	<0.05
BMI (kg/m^2^, mean ± SD)	25.5 ± 3.1	26.7 ± 3.6	t(272) = 1.80	0.07
* ASA ≥ III n (%)	12 (17%)	66 (33%)	χ^2^(1) = 6.01	<0.05
* Prior abdominal surgery n (%)	8 (11%)	42 (21%)	χ^2^(1) = 3.52	0.06
* Multivisceral resection n (%)	0 (0%)	19 (9%)	Fisher’s Exact	<0.01
* Tumor size (cm, mean ± SD)	4.5 ± 1.8	5.3 ± 2.1	t(272) = 2.40	0.02
Tumor location (8 regions)	–	–	χ^2^(7) = 44.11	<0.001
Tumor staging (Tis–T4b)	–	–	χ^2^(5) = 16.72	<0.01
Lymph nodes retrieved (mean ± SD)	16.1 ± 5.7	19.7 ± 10.6	Wilcoxon rank-sum (W = 8239.5)	<0.05
N positive nodes (%)	24 (34%)	86 (42%)	χ^2^(1) = 1.28	0.26
Margin distance (mm, mean ± SD)	5.7 ± 3.1	4.8 ± 4.5	Wilcoxon rank-sum (W = 5074.5)	<0.01
Positive margin n (%)	0 (0%)	11 (5%)	Fisher’s Exact	0.07
Length of hospital stay (days, mean ± SD)	7.1 ± 2.3	13.2 ± 6.8	Wilcoxon rank-sum (W = 12,354)	<0.001
* 30-day mortality n (%)	0 (0%)	2 (1%)	Fisher’s Exact	0.32
* Conversion to open n (%)	0 (0%)	–	–	–

Note. LS = laparoscopic surgical procedure; OS = open surgical procedure; MSD = standard deviation. ASA = American Society of Anesthesiologists. p-values are two-tailed. Categorical variables analyzed with χ^2^ or Fisher’s exact tests; continuous variables analyzed with Wilcoxon rank-sum tests due to non-normal distributions. * Tumor invasion data were incomplete because 3 LS cases lacked recorded pathology details; thus, only 68/71 LS patients were evaluable for this variable. Positive—invasion of the resection margin; Negative—no invasion of the resection margin.

## Data Availability

The data is available on demand from the corresponding author.

## Abbreviations

The following abbreviations are used in this manuscript:

LS	Laparoscopy
CRM	Circumferential resection margin
OS	Open surgery
BMI	Body mass index

## References

[1] Jacobs M., Verdeja J.C., Goldstein H.S. (1991). Minimally invasive colon resection (laparoscopic colectomy). Surg. Laparosc. Endosc..

[2] Kuhry E., Schwenk W., Gaupset R., Romild U., Bonjer H.J., Cochrane Colorectal Cancer Group (2008). Long-term results of laparoscopic colorectal cancer resection. Cochrane Database Syst. Rev..

[3] Lacy A.M., García-Valdecasas J.C., Delgado S., Castells A., Taule J., Piqué J.M. (2002). Laparoscopy-assisted colectomy versus open colectomy for non-metastatic colon cancer: A randomised trial. Lancet.

[4] Theophilus M., Platell C., Spilsbury K., Phillips D. (2014). Long-term survival following laparoscopic and open colectomy for colon cancer: Meta-analysis of randomized controlled trials. Color. Dis..

[5] Devoto L., Celentano V., Cohen R., Khan J., Chand M. (2017). Colorectal cancer surgery in the very elderly patient: A systematic review of laparoscopic versus open colorectal resection. Int. J. Color. Dis..

[6] Shah P.R., Joseph A., Haray P.N. (2005). Laparoscopic colorectal surgery: Learning curve and training implications. Postgrad. Med. J..

[7] Tekkis P.P., Senagore A.J., Delaney C.P., Fazio V.W. (2005). Evaluation of the learning curve in laparoscopic colorectal surgery: Comparison of right-sided and left-sided resections. Ann. Surg..

[8] Bonjer H.J., Deijen C.L., Abis G.A., Cuesta M.A., van der Pas M.H., de Lange-de Klerk E.S., Lacy A.M., Bemelman W.A., Andersson J., Angenete E. (2015). A randomized trial of laparoscopic versus open surgery for rectal cancer. N. Engl. J. Med..

[9] Gietelink L., Wouters M.W., Bemelman W.A., Dekker J.W., Tollenaar R.A., Tanis P.J., Dutch Surgical Colorectal Cancer Audit Group (2016). Reduced 30-Day Mortality After Laparoscopic Colorectal Cancer Surgery: A Population-Based Study From the Dutch Surgical Colorectal Audit (DSCA). Ann. Surg..

[10] Warps A.K., Saraste D., Westerterp M., Detering R., Sjövall A., Martling A., Dekker J.W.T., Tollenaar R.A.E.M., Matthiessen P., Tanis P.J. (2022). National differences in implementation of minimally invasive surgery for colorectal cancer and the influence on short-term outcomes. Surg. Endosc..

[11] Morton A.J., Simpson A., Humes D.J. (2023). Regional variations and deprivation are linked to poorer access to laparoscopic and robotic colorectal surgery: A national study in England. Tech. Coloproctol..

[12] Coleman M.G., Hanna G.B., Kennedy R., National Training Programme (Lapco) (2011). The National Training Programme for Laparoscopic Colorectal Surgery in England: A new training paradigm. Color. Dis..

[13] Taylor E.F., Thomas J.D., Whitehouse L.E., Quirke P., Jayne D., Finan P.J., Forman D., Wilkinson J.R., Morris E.J. (2013). Population-based study of laparoscopic colorectal cancer surgery 2006–2008. Br. J. Surg..

[14] Ghadban T., Reeh M., Bockhorn M., Heumann A., Grotelueschen R., Bachmann K., Izbicki J.R., Perez D.R. (2018). Minimally invasive surgery for colorectal cancer remains underutilized in Germany despite its nationwide application over the last decade. Sci. Rep..

[15] Bintintan V.V., Fagarasan V., Seicean R.I., Andras D., Ene A.I., Chira R., Bintintan A., Nagy G., Petrisor C., Cocu S. (2025). Laparoscopic Radical Colectomy with Complete Mesocolic Excision Offers Similar Results Compared with Open Surgery. Medicina.

[16] Negrut R.L., Cote A., Caus V.A., Maghiar A.M. (2024). Systematic Review and Meta-Analysis of Laparoscopic versus Robotic-Assisted Surgery for Colon Cancer: Efficacy, Safety, and Outcomes-A Focus on Studies from 2020-2024. Cancers.

[17] Morarasu S., Clancy C., Gorgun E., Yilmaz S., Ivanecz A., Kawakatsu S., Musina A.M., Velenciuc N., Roata C.E., Dimofte G.M. (2023). Laparoscopic versus open resection of primary colorectal cancers and synchronous liver metastasis: A systematic review and meta-analysis. Int. J. Color. Dis..

[18] Shah S. (2025). Simplified and reproducible laparoscopic complete mesocolic excision with D3 right hemicolectomy. Color. Dis..

[19] Pedrazzani C., Conti C., Zamboni G.A., Chincarini M., Turri G., Valdegamberi A., Guglielmi A. (2020). Impact of visceral obesity and sarcobesity on surgical outcomes and recovery after laparoscopic resection for colorectal cancer. Clin. Nutr..

[20] Chiu C.C., Lin W.L., Shi H.Y., Huang C.C., Chen J.J., Su S.B., Lai C.C., Chao C.M., Tsao C.J., Chen S.H. (2019). Comparison of Oncologic Outcomes in Laparoscopic versus Open Surgery for Non-Metastatic Colorectal Cancer: Personal Experience in a Single Institution. J. Clin. Med..

[21] Edge S.B., Compton C.C. (2010). The American Joint Committee on Cancer: The 7th edition of the AJCC cancer staging manual and the future of TNM. Ann. Surg. Oncol..

[22] Szostek J., Serafin M., Mąka M., Jabłońska B., Mrowiec S. (2025). Right- vs left-sided colon cancer: 5-year single-centre observational study. Cancers.

[23] Ghioldis A.C., Sarbu V., Dan C., Butelchin C., Olteanu C., Popescu R.C. (2024). Open versus laparoscopic surgery for rectal cancer in elderly patients with comorbidities. Arch. Balk. Med. Union..

[24] Lee L., Erkan A., Alhassan N., Kelly J.J., Nassif G.J., Albert M.R., Monson J.R. (2018). Lower survival after right- vs left-sided colon cancers: Is extended lymphadenectomy the answer?. Surg. Oncol..

[25] Balciscueta Z., Balciscueta I., Uribe N., Pellino G., Frasson M., García-Granero E., Garcia-Granero A. (2021). D3-lymphadenectomy enhances oncological clearance in right colon cancer: Meta-analysis. Eur. J. Surg. Oncol..

[26] Bernhoff R., Sjövall A., Buchli C., Granath F., Holm T., Martling A. (2018). Complete mesocolic excision in right-sided colon cancer does not increase severe short-term postoperative adverse events. Color. Dis..

[27] He L.H., Yang B., Su X.Q., Zhou Y., Zhang Z. (2022). Comparison of clinical efficacy and postoperative inflammatory response between laparoscopic and open radical resection of colorectal cancer. World J. Clin. Cases.

[28] Macovei Oprescu A.M., Dumitriu B., Stefan M.A., Oprescu C., Venter D.P., Mircea V., Valcea S. (2024). Open Versus Laparoscopic Oncological Resections for Colon Cancer: An Experience at an Average-Volume Center. Cureus.

[29] Enciu O., Avino A., Calu V., Toma E.A., Tulin A., Tulin R., Slavu I., Răducu L., Balcangiu-Stroescu A.E., Gheoca Mutu D.E. (2022). Laparoscopic vs open resection for colon cancer-quality of oncologic resection evaluation in a medium volume center. Exp. Ther. Med..

[30] Park J.S., Huh J.W., Park Y.A., Cho Y.B., Yun S.H., Kim H.C., Lee W.Y., Chun H.K. (2016). Clinically suspected T4 colorectal cancer may be resected laparoscopically. BMC Cancer.

[31] Braga M., Vignali A., Zuliani W., Frasson M., Di Serio C., Di Carlo V. (2005). Laparoscopic vs open colorectal surgery: Cost-benefit analysis in a randomized trial. Ann. Surg..

[32] Wei D., Johnston S., Goldstein L., Nagle D. (2020). Minimally invasive colectomy is associated with reduced risk of anastomotic leak and other major perioperative complications and reduced hospital resource utilization as compared with open surgery: A retrospective population-based study of comparative effectiveness and trends of surgical approach. Surg. Endosc..

[33] American Joint Committee on Cancer (AJCC) (2018). AJCC 8th Edition Colon and Rectal Cancer Staging: Colon & Rectal—Webinar/Presentation. https://www.facs.org/media/vf4n5vzd/colorectal-8th-ed.pdf.

[34] Huang F., Jiang S., Wei R., Xiao T., Wei F., Zheng Z., Liu Q. (2024). Association of resection margin distance with anastomotic recurrence in stage I-III colon cancer: Data from the National Colorectal Cancer Cohort (NCRCC) study in China. Int. J. Color. Dis..

[35] Gkionis I.G., Flamourakis M.E., Tsagkataki E.S., Kaloeidi E.I., Spiridakis K.G., Kostakis G.E., Alegkakis A.K., Christodoulakis M.S. (2020). Multidimensional analysis of the learning curve for laparoscopic colorectal surgery in a regional hospital: The implementation of a standardized surgical procedure counterbalances the lack of experience. BMC Surg..

[36] Jayne D., Pigazzi A., Marshall H., Croft J., Corrigan N., Copeland J., Quirke P., West N., Rautio T., Thomassen N. (2017). Effect of robotic-assisted vs conventional laparoscopic surgery on risk of conversion to open surgery among patients undergoing resection for rectal cancer. JAMA.

[37] Miskovic D., Ni M., Wyles S.M., Tekkis P., Hanna G.B. (2012). Learning curve and case selection in laparoscopic colorectal surgery: Systematic review and international multicenter analysis of 4852 cases. Dis. Colon Rectum.

[38] Hanna G.B., Mackenzie H., Miskovic D., Ni M., Wyles S., Aylin P., Parvaiz A., Cecil T., Gudgeon A., Griffith J. (2022). Laparoscopic colorectal surgery outcomes improved after National Training Program (LAPCO) for specialists in England. Ann. Surg..

[39] Xynos E., Gouvas N., Triantopoulou C., Tekkis P., Vini L., Tzardi M., Boukovinas I., Androulakis N., Athanasiadis A., Christodoulou C. (2016). Clinical practice guidelines for the surgical management of colon cancer: A consensus statement of the Hellenic and Cypriot Colorectal Cancer Study Group by the HeSMO. Ann. Gastroenterol..

[40] Kolfschoten N.E., Van Leersum N.J., Gooiker G.A., Van De Mheen P.J., Eddes E.H., Kievit J., Brand R., Tanis P.J., Bemelman W.A., Tollenaar R.A. (2013). Successful and safe introduction of laparoscopic colorectal cancer surgery in Dutch hospitals. Ann. Surg..

[41] Odermatt M., Khan J., Parvaiz A. (2022). Supervised training of laparoscopic colorectal cancer resections does not adversely affect short- and long-term outcomes: A propensity-score-matched cohort study. World J. Surg. Oncol..

[42] Bertelsen C.A., Neuenschwander A.U., Jansen J.E., Wilhelmsen M., Kirkegaard-Klitbo A., Tenma J.R., Bols B., Ingeholm P., Rasmussen L.A., Jepsen L.V. (2015). Disease-free survival after complete mesocolic excision compared with conventional colon cancer surgery: A retrospective, population-based study. Lancet Oncol..

[43] Mackenzie H., Miskovic D., Ni M., Parvaiz A., Acheson A.G., Jenkins J.T., Griffith J., Coleman M.G., Hanna G.B. (2013). Clinical and educational proficiency gain of supervised laparoscopic colorectal surgical trainees. Surg. Endosc..

[44] Akiyoshi T., Kuroyanagi H., Ueno M., Oya M., Fujimoto Y., Konishi T., Yamaguchi T. (2011). Learning curve for standardized laparoscopic surgery for colorectal cancer under supervision: Single-center experience. Surg. Endosc..

[45] Staiger R.D., Rössler F., Kim M.J., Brown C., Trenti L., Sasaki T., Uluk D., Campana J.P., Giacca M., Schiltz B. (2022). Benchmarks in colorectal surgery: Multinational study to define quality thresholds in anterior resections. Br. J. Surg..

[46] Massa I., Ghignone F., Ugolini G., Ercolani G., Montroni I., Capelli P., Garulli G., Catena F., Lucchi A., Ansaloni L. (2022). Emilia-Romagna Surgical Colorectal Cancer Audit (ESCA): A value-based healthcare retro-prospective study to measure and improve the quality of surgical care in colorectal cancer. Int. J. Color. Dis..

[47] Shah P.M., Johnston L., Sarosiek B., Rogers T., Cohn H., Hedrick T.L. (2017). Reducing readmissions while shortening length of stay: The positive impact of an enhanced recovery protocol in colorectal surgery. Dis. Colon. Rectum..

[48] Fleshman J., Branda M., Sargent D.J., Boller A.M., George V., Abbas M., Peters W.R., Maun D., Chang G., Herline A. (2015). Effect of laparoscopic-assisted vs open resection of stage II/III rectal cancer on pathologic outcomes: ACOSOG Z6051. JAMA.

[49] Kitano S., Inomata M., Sato A., Yoshimura K., Moriya Y. (2005). Randomized controlled trial to evaluate laparoscopic surgery for colorectal cancer: Japan Clinical Oncology Group Study JCOG 0404. Jpn. J. Clin. Oncol..

[50] Kang S.B., Park J.W., Jeong S.Y., Nam B.H., Choi H.S., Kim D.W., Lim S.B., Lee T.G., Kim D.Y., Kim J.S. (2010). Open versus laparoscopic surgery for mid or low rectal cancer after neoadjuvant chemoradiotherapy (COREAN trial): Short-term outcomes of an open-label randomised controlled trial. Lancet Oncol..

[51] Deijen C.L., Vasmel J.E., de Lange-de Klerk E.S., Cuesta M.A., Coene P.P., Lange J.F., Meijerink W.J., Jakimowicz J.J., Jeekel J., Kazemier G. (2017). Ten-year outcomes of a randomised trial of laparoscopic vs open surgery for colon cancer. Surg. Endosc..

[52] Kitano S., Inomata M., Mizusawa J., Katayama H., Watanabe M., Yamamoto S., Ito M., Saito S., Fujii S., Konishi F. (2017). Survival outcomes following laparoscopic versus open D3 dissection for stage II or III colon cancer (JCOG0404): A phase 3 randomized controlled trial. Lancet Gastroenterol. Hepatol..

[53] Vitous C.A., Ivatury S.J., Suwanabol P.A. (2021). Value of Qualitative Research in Colorectal Surgery. Dis. Colon. Rectum..

[54] van der Pas M.H., Haglind E., Cuesta M.A., Fürst A., Lacy A.M., Hop W.C., Bonjer H.J., COLOR II Study Group (2013). Laparoscopic versus open surgery for rectal cancer (COLOR II): Short-term outcomes of a randomized, phase 3 trial. Lancet Oncol..

